# Editorial: Exploring novel targets and therapies to combat antimicrobial resistance

**DOI:** 10.3389/fmicb.2025.1642199

**Published:** 2025-06-19

**Authors:** Karina Tuz, Oscar X. Juárez, Corina D. Ceapă

**Affiliations:** ^1^Department of Biological Sciences, Illinois Institute of Technology, Chicago, IL, United States; ^2^Laboratory of Microbiology, Chemistry Institute, Universidad Nacional Autónoma de México, Mexico City, Mexico

**Keywords:** antimicrobial resistance, antimicrobials, repurposing of antibiotics, *Drosophila melanogaster* model, adjuvants

Antimicrobial resistance is one of the grand challenges of our time, posing an important public health problem that threatens modern medicine. The contributing authors of this topic have uncovered novel antibiotic mechanisms, antimicrobial peptides from uncommon sources, described new avenues for the development of adjuvants, repurposed existing antibiotics, created chimeric enzymes with enhanced antimicrobial activity and have provided insight into a novel animal model that could reduce both time and costs for drug discovery, all these different approaches and breakthroughs are a testament of the human imagination that can could lead to the development of new antimicrobial therapies and treatments.

The work by Ye et al. improves our understanding of the effects of β-lactam antibiotics, such as ceftazidime, ampicillin, and meropenem on *Escherichia coli* metabolism and oxidative stress. Their results show a downregulation of essential energy metabolites and reduced antioxidant metabolites upon antibiotic treatment, suggesting that oxidative stress produced by these antibiotics may overwhelm bacterial defenses. The extensive metabolic disturbances linked to reactive oxygen species (ROS) production by β-lactam antibiotics, potentially compromise vital cellular components. By revealing how β-lactam antibiotics cause metabolic disturbances and ROS production, this study opens possibilities for developing therapies that target these metabolic pathways to enhance antibiotic efficacy and combat resistance. Also, it serves as an important reminder that non-target effects can complement the actions of antibiotics.

PmrC is an enzyme that transfers phosphoethanolamine to LPS, which confers colistin resistance to *Acinetobacter baumannii*. This mechanism is of outermost importance since colistin is a last-line antibiotic against *A. baumannii*. Romano et al. identified the compound s-Phen, through virtual screening of the PmrC enzyme after biochemical characterization, demonstrating its potential to synergistically reduce colistin resistance in *A. baumannii* with no toxicity. This study highlights PmrC as a crucial drug target and s-Phen as a promising adjuvant to combat multidrug-resistant pathogens. The findings of this study could significantly impact the development of new antimicrobial therapies and improve treatment outcomes for infections caused by multidrug-resistant pathogens.

The study by O'Connor et al. represents the first bacteriocin screening from canine commensal sources, confirming this environment is rich in bacteriocin-producing strains. This work identifies and characterizes caledonicin, a novel bacteriocin from *Staphylococcus caledonicus*. The work is a nice example of isolation of antimicrobial-producing bacteria, antibacterial activity assessment by deferred antagonism, bacteriocin mining through WGS, and bioinformatics analysis. They propose bacteriocins as alternatives or complements to traditional antibiotics, emphasizing their potential application in veterinary medicine, particularly for domestic animals like dogs.

Similarly, the study by Sun et al. identifies molecules from other animal sources, such as the fly (*Musca domestica*), where they identified the peptide AMP-17 with activity against *Candida albicans*, an opportunistic fungus that causes nosocomial infections that can develop into deadly systemic infections. The peptide has similar activity to fuconazole, a standard drug used in *Candida* treatment. This study underscores the potential of peptides to treat emerging infectious diseases.

Ferreira et al.'s investigation focuses on *Staphylococcus aureus*, a pathogen of paramount importance in hospital-acquired infections. This microorganism is frequently isolated from clinical samples and is well-recognized for its remarkable ability to form biofilms, which greatly complicates treatments. Moreover, growing concerns regarding the increasing prevalence of methicillin-resistant *S. aureus* (MRSA) strains underscore the critical necessity for advanced research in this area. *S. aureus* can colonize medical devices during implantation procedures and during bacteremia, which challenges treatment. Effective treatment requires aggressive antibiotic treatment with high biofilm penetration. Rifabutin, a structural analog to rifampicin, is a drug approved to treat tuberculosis and prevent dissemination of *Mycobacterium avium* complex in patients with advanced HIV. The authors tested rifabutin on clinical isolates and found that biofilms are susceptible to low concentration of rifabutin. These results make the repurposing of rifabutin plausible and highlight the importance of identifying new uses for already approved drugs which are safe and offer a potentially faster and cheaper approach to drug discovery.

Wang et al. created a chimeric endolysin for the treatment of mastitis induced by streptococci. Endolysins, derived from bacteriophages, are enzymes with lytic activity on the bacterial cell wall, and had the advantage of low resistance development. Endolysins have a modular structure that the authors exploited to construct a chimeric peptide with enhanced bactericidal activity against Streptococci species. The chimera Cly2v consisted of a catalytic domain and a cell binding domain, both from different bacteriophages, which has a stronger activity than the parental endolysin. Even though the chimeric endolysin does not completely clear biofilm nor the bactericidal effect is complete, the survival rate increased considerably on an animal model of infection with *S. agalactiae* after Cly2v treatment. This is a significant advancement in the control of the disease since bovine mastitis has considerable economic loses and poses zoonotic infection risks.

Finally, new treatment research requires compound characterization at all levels from molecular (structure, mechanism of action, metabolism) to organismal levels (pharmacokinetics, pharmacodynamics). Screening of compounds is time consuming, expensive and raises ethical concerns for the use of mammalian animal models. The review by Vidal et al., postulates *Drosophila melanogaster* as an intermediate model in drug discovery that could be important for toxicity studies during preclinical findings. The authors highlight that this model organism is not only cost-effective but could reduce the use of vertebrates in preclinical development, as many low-quality compounds need to be tested as we face the extraordinary challenge of discovery and development of new antibiotics. The authors emphasize that this model has been used for human diseases and has many advantages such as small size, short life-cycle, large number of offspring, low-cost husbandry and a fully sequenced genome. The *D. melanogaster* model is discussed in comparison to other invertebrate models such as *Caenorhabditis elegans* and *Galleria mellonella*, covering the pros and cons in each case, and the paper is undoubtedly an excellent tool to select a testing system.

These diverse investigations highlight the various strategies being employed to tackle the growing challenge of antimicrobial resistance, paving the way for safer and more effective therapeutic strategies. This marks a promising advancement in the fight against multidrug-resistant infections.

